# Supplementary material for: Urinary Comprehensive Genomic Profiling Correlates Urothelial Carcinoma Mutations with Clinical Risk and Efficacy of Intervention
Source: J Clin Med. 2022 Sep 30;11(19):5827. doi: 10.3390/jcm11195827 (PMC9571552; doi:10.3390/jcm11195827)
Supplement: Supplementary file 1 [file jcm-11-05827-s001.zip › jcm-1875416-supplementary.pdf]

Journal of
Clinical Medicine

MDPI

Supplementary Materials

Urinary Comprehensive Genomic Profiling Correlates Urothelial Carcinoma Mutations with Clinical Risk and Efficacy of Intervention

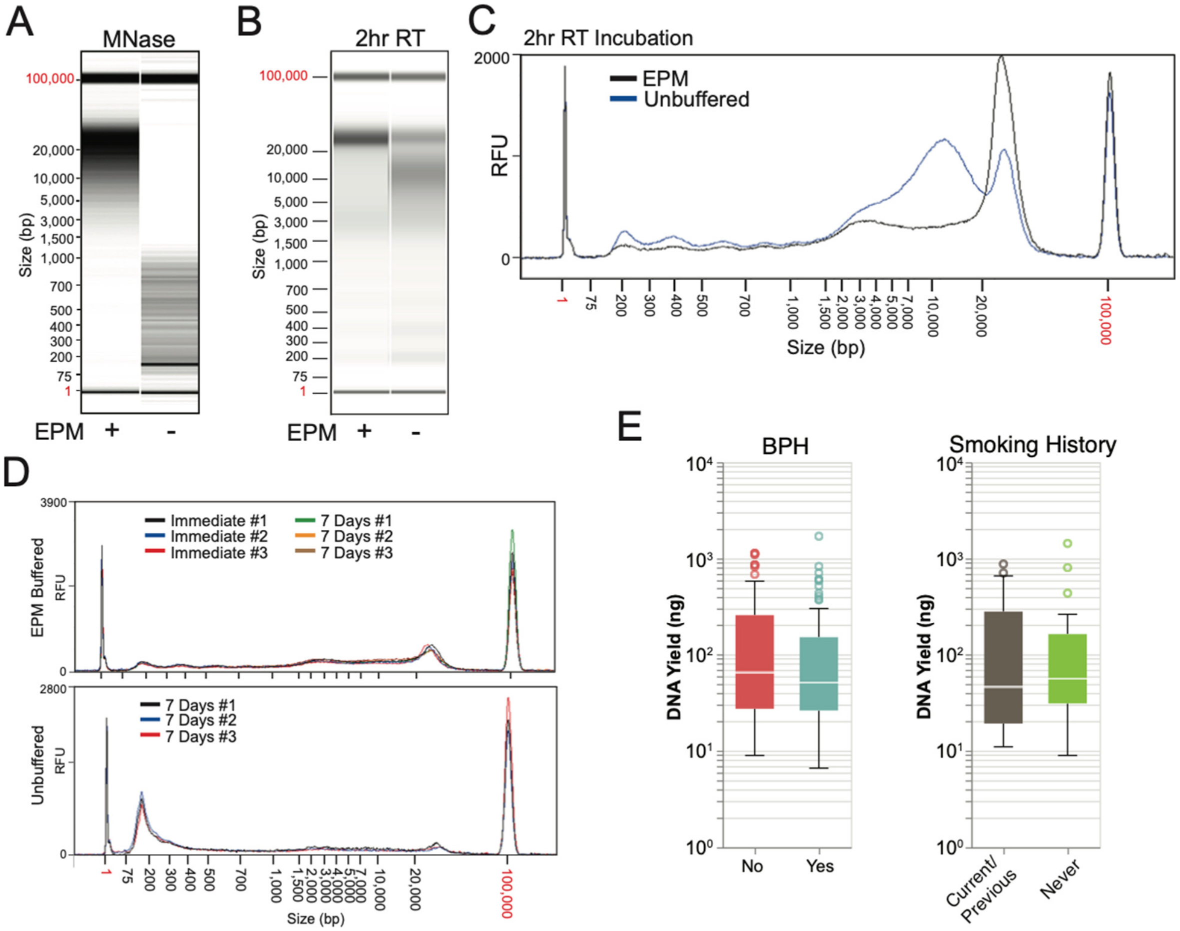

Figure S1. UroAmplitude test procedure development. (**A**) Fragment analyzer (FA) gel view of purified DNA incubated with micrococcal nuclease (MNase) in the presence (+) or absence (−) of Enhanced Preservation Media (EPM). (**B**,**C**) FA gel and electrophoretogram view of purified DNA following 2 h room temperature incubation in the presence (+) or absence (−) of EPM. (**D**) FA electrophoretogram profiles of purified DNA following either immediate or 7 day incubation with EPM (top panel) or without EPM (bottom panel). Each incubation and extraction is performed in triplicate. (**E**) Median DNA extraction yields among individuals with and without benign prostatic hyperplasia (BPH) (n = 130, left panel) and among ever and never smokers (n = 33, right panel).

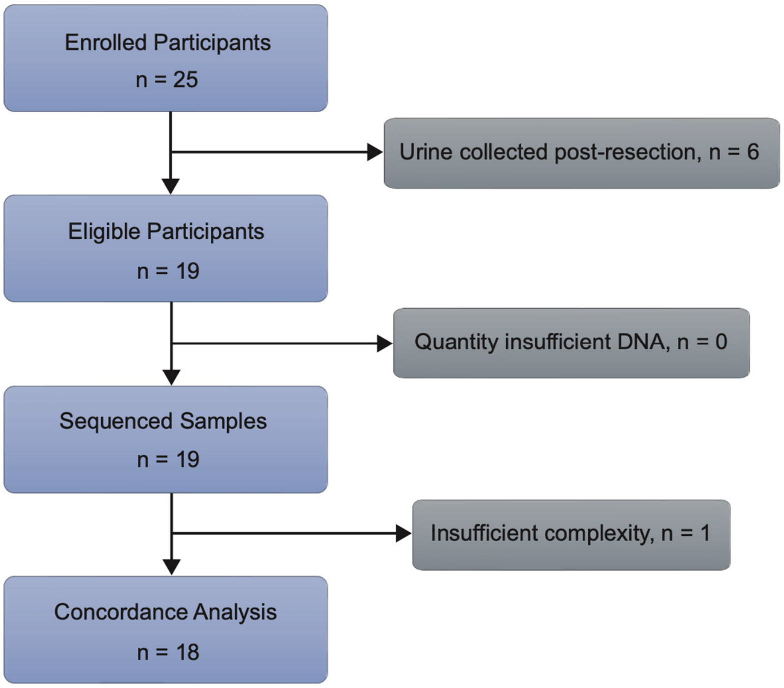

Figure S2. STARD diagram of prospective concordance analysis. Documentation of samples excluded from concordance analysis and justification.

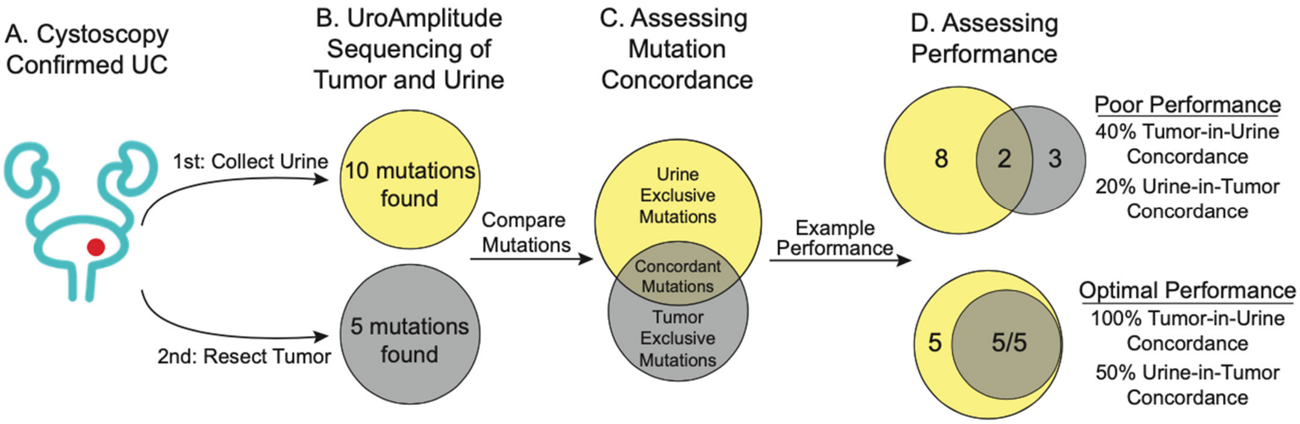

Figure S3. Experimental design for assessing mutational concordance between urine and tumor. (**A**,**B**) Matched tumor and urine samples are analyzed by UroAmplitude. In this example, we find ten mutations in the urine and five mutations in the tumor. Due to natural bladder clonality observed with age, and the likelihood of pre-cancerous, secondary lesions present elsewhere in the bladder, more mutations are expected to be found in the urine than the tumor. (**C**,**D**) Mutations found in the tumor and urine are compared to identify urine-exclusive events, tumor-exclusive events, and concordant events. In the optimal assay, all five tumor mutations are found in the urine (100% tumor-in-urine concordance) providing perfect comprehensive genomic profiling of the tumor from urine. Urine-exclusive mutations provide insight into bladder health beyond the tumor, and risk assessment can be made based on mutation type and allele frequency.

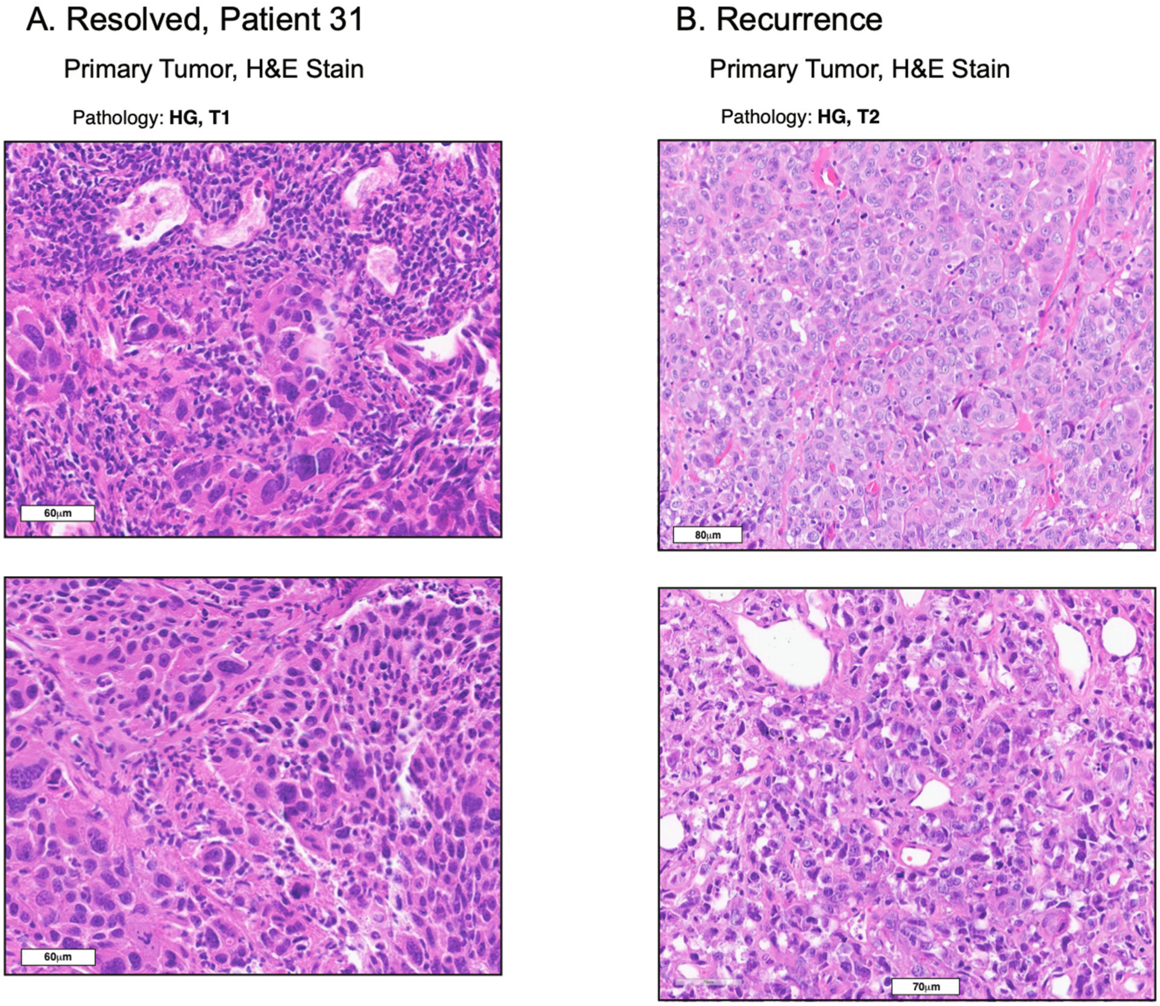

Figure S4. Independent pathological assessment of UC tumors. FFPE sample sections were stained with haematoxylin and eosin (H&E) and reviewed by a second independent pathologist to assess tumor grading.

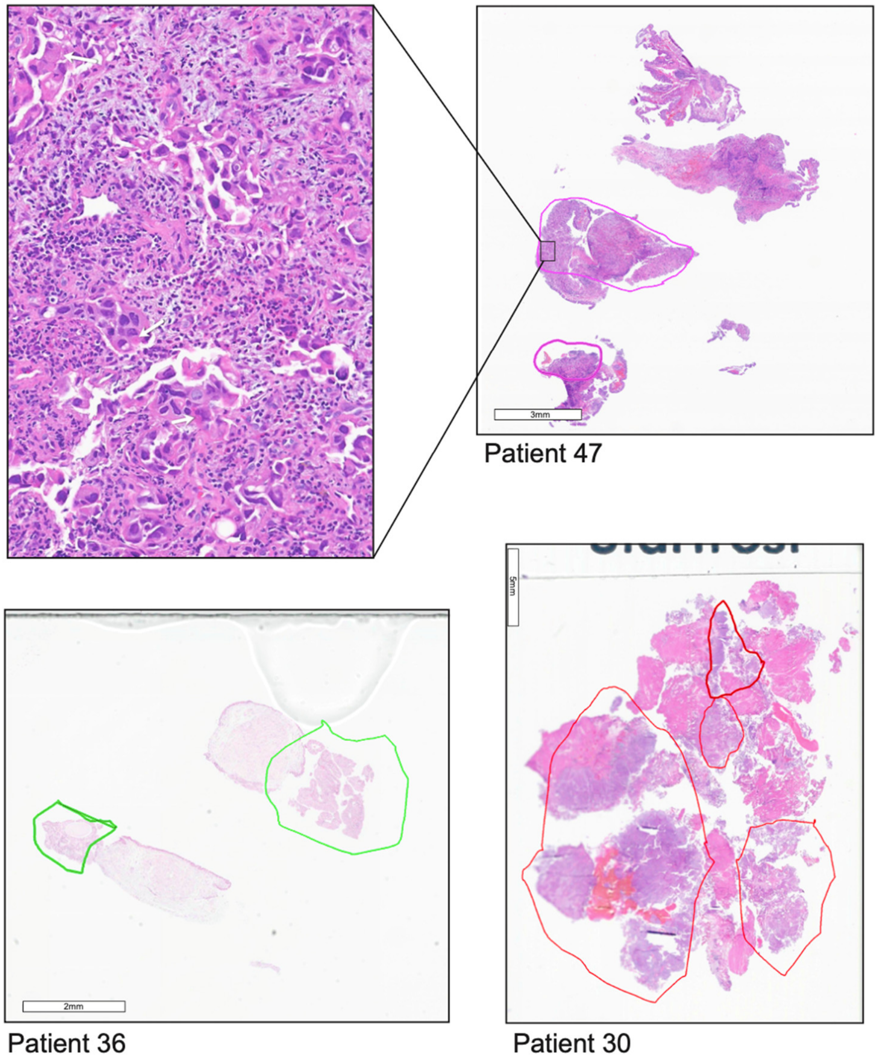

Figure S5. Preparation of FFPE tumor sample sections for DNA extraction. Slides with minority percentages of tumor tissue were annotated to highlight tumor and healthy tissue was removed by microdissection prior to DNA extraction.

Table S1. Cohort Clinical Characteristics.

Cohort Clinical Characteristics								Analysis			
								Mutation			Process
ID	Sample	Source	Disease Status	Stage	Grade	Age	Sex	Longi-tudi-nal	Onco-map	Concor-dance	Extract-ion
1	cgi_128	urine	negative	t3, cis	hg	69	m	0	0	0	1
2	cgi_127	urine	positive		hg	80	m	0	0	0	1
2	cgi_130	urine	positive		hg	80	m	0	0	0	1
2	cgi_131	urine	positive		hg	80	m	0	0	0	1
2	cgi_132	urine	positive		hg	80	m	0	0	0	1
2	cgi_158	urine	positive		hg	80	m	0	0	0	1
2	cgi_136	urine	positive		hg	80	m	0	0	0	1
3	cgi_122	urine	negative			87	m	0	0	0	1
3	cgi_172	urine	negative			87	m	0	0	0	1
3	cgi_167	urine	negative			87	m	0	0	0	1

3	cgi_185	urine	negative	t1	hg	88	m	0	0	0	1
3	cgi_183	urine	negative	t1	hg	87	m	0	0	0	1
3	cgi_133	urine	negative			87	m	0	0	0	1
3	cgi_123	urine	negative			87	m	0	0	0	1
4	cgi_234	urine	negative			61	m	0	0	0	1
6	cgi_073	urine	negative			93	f	1	0	0	0
6	cgi_072	urine	negative			93	f	1	0	0	0
6	cgi_071	urine	negative			93	f	1	0	0	0
6	cgi_070	urine	positive	cis	hg	93	f	1	0	0	1
6	cgi_088	urine	positive	t1	hg	93	f	1	0	0	0
6	cgi_074	urine	negative			93	f	1	0	0	0
7	cgi_253	urine	negative			67	f	0	0	0	1
7	cgi_250	urine	negative			67	f	0	0	0	1
9	cgi_438	urine	negative			86	m	0	0	0	1
10	cgi_076	urine	positive		hg	79	m	1	0	0	1
10	cgi_078	urine	negative		hg	79	m	1	0	0	0
10	cgi_079	urine	negative		hg	79	m	1	0	0	0
10	cgi_254	urine	negative			79	m	0	0	0	1
10	cgi_075	urine	positive		hg	79	m	1	0	0	1
10	cgi_252	urine	negative			79	m	0	0	0	1
10	cgi_245	urine	positive			79	m	0	0	0	1
10	cgi_251	urine	negative			79	m	0	0	0	1
10	cgi_249	urine	negative			79	m	0	0	0	1
10	cgi_077	urine	positive		hg	79	m	1	0	0	1
10	cgi_080	urine	positive	ta,cis	hg	79	m	1	0	0	1
11	cgi_194	urine	negative			86	f	0	0	0	1
11	cgi_212	urine	negative			87	f	0	0	0	1
11	cgi_220	urine	negative		hg	87	f	0	0	0	1
12	cgi_218	urine	positive	cis	hg	94	f	0	0	0	1
12	cgi_210	urine	negative	cis	hg	94	f	0	0	0	1
12	cgi_213	urine	negative	cis	hg	94	f	0	0	0	1
13	cgi_087	urine	positive	cis	hg	70	m	1	0	0	1
13	cgi_091	urine				70	m	1	0	0	0
13	cgi_090	urine				70	m	1	0	0	0
13	cgi_089	urine				70	m	1	0	0	0
13	cgi_085	urine	positive	cis	hg	70	m	1	0	0	1
13	cgi_086	urine				70	m	1	0	0	0
13	cgi_082	urine	negative	cis	hg	70	m	1	0	0	0
13	cgi_083	urine				70	m	1	0	0	0
13	cgi_081	urine	positive	cis	hg	70	m	1	0	0	0
13	cgi_084	urine				70	m	1	0	0	0
14	cgi_207	urine	negative			83	f	0	0	0	1
14	cgi_195	urine	negative			83	f	0	0	0	1
14	cgi_216	urine	negative	ta	lg	83	f	0	0	0	1
15	cgi_161	urine	negative			98	m	0	0	0	1
15	cgi_166	urine	negative			98	m	0	0	0	1
16	cgi_143	urine	positive	t1	hg	89	f	0	0	0	1
16	cgi_134	urine	positive	t1	hg	89	f	0	0	0	1
17	cgi_255	urine	negative	ta	hg	77	m	0	0	0	1

17	cgi_247	urine	positive	ta	lg	77	m	0	0	0	1
17	cgi_236	urine	positive	ta	lg	76	m	0	0	0	1
18	cgi_235	urine	positive	ta	lg	69	m	0	0	0	1
18	cgi_248	urine	positive	ta	lg	70	m	0	0	0	1
19	cgi_364	urine	positive	ta	lg	67	f	0	0	0	1
19	cgi_290	urine	positive	ta	lg	67	f	0	0	0	1
20	cgi_385	urine	positive	ta	hg	77	m	0	0	0	1
20	cgi_003	tumor	positive	ta	hg	77	m	0	1	1	0
20	cgi_004	urine	positive	ta	hg	77	m	0	1	1	0
20	cgi_005	urine	negative	ta	hg	77	m	0	1	0	0
21	cgi_332	urine	positive	ta	lg	72	m	0	0	0	1
21	cgi_266	urine	positive	ta	lg	71	m	0	0	0	1
21	cgi_391	urine	positive	ta	lg	72	m	0	0	0	1
22	cgi_008	urine	positive	ta	lg	64	m	1	1	0	0
22	cgi_007	tumor	positive	ta	lg	64	m	1	1	1	0
22	cgi_010	urine	negative	ta	lg	65	m	1	1	0	1
22	cgi_009	urine	negative	ta	lg	65	m	1	1	0	1
22	cgi_011	urine	negative	ta	lg	65	m	1	1	0	1
22	cgi_006	urine	positive	ta	lg	64	m	0	1	1	1
24	cgi_141	urine	positive	ta	lg	58	m	0	0	0	1
24	cgi_146	urine	negative	ta	lg	58	m	0	0	0	1
26	cgi_014	tumor	positive	ta	lg	64	f	0	1	1	0
26	cgi_015	urine	negative	ta	lg	64	f	0	1	0	1
26	cgi_013	urine	positive	ta	lg	64	f	0	1	1	1
27	cgi_406	urine	negative			78	m	0	0	0	1
27	cgi_338	urine	positive	ta	lg	77	m	0	0	0	1
28	cgi_397	urine	positive	ta	hg	77	f	0	0	0	1
28	cgi_277	urine	positive	ta	hg	76	f	0	0	0	1
29	cgi_322	urine	positive	ta	lg	81	m	0	0	0	1
29	cgi_350	urine	positive	cis	hg	81	m	0	0	0	1
30	cgi_016	urine	positive	t4	hg	75	m	1	1	0	1
30	cgi_019	urine	positive	t4	hg	75	m	0	1	1	1
30	cgi_017	tumor	positive	t4	hg	75	m	1	1	1	0
31	cgi_023	urine	negative	ta	hg	84	m	1	1	0	1
31	cgi_021	urine	negative	ta	hg	84	m	1	1	0	0
31	cgi_020	tumor	positive	ta	hg	84	m	1	1	0	0
31	cgi_022	urine	negative	ta	hg	84	m	1	1	0	1
32	cgi_269	urine	negative	ta	hg	83	m	0	0	0	1
32	cgi_396	urine	negative			84	m	0	0	0	1
33	cgi_339	urine	negative		hg	67	m	0	0	0	1
33	cgi_404	urine	negative		hg	97	m	0	0	0	1
33	cgi_393	urine	negative		hg	67	m	0	0	0	1
33	cgi_275	urine	negative	t1	lg	67	m	0	0	0	1
34	cgi_389	urine	negative	cis	hg	59	m	0	0	0	1
34	cgi_405	urine	negative	cis	hg	59	m	0	0	0	1
34	cgi_337	urine	negative	cis	hg	59	m	0	0	0	1
34	cgi_264	urine	negative	cis	hg	58	m	0	0	0	1
34	cgi_302	urine	negative	cis	hg	59	m	0	0	0	1
35	cgi_025	urine	positive	ta	lg	92	f	0	1	1	1

35	cgi_024	tumor	positive	ta	lg	92	f	0	1	1	0
35	cgi_098	urine	negative	ta	lg	92	f	0	1	0	1
35	cgi_026	urine	negative	ta	lg	92	f	0	1	0	1
36	cgi_094	urine	negative	ta	lg	84	m	0	1	0	1
36	cgi_028	urine	negative	ta	lg	84	m	0	1	0	1
36	cgi_027	tumor	positive	ta	lg	84	m	0	1	0	0
37	cgi_381	urine	negative	ta	lg	71	m	0	0	0	1
37	cgi_270	urine	negative	ta	lg	71	m	0	0	0	1
38	cgi_032	urine	negative	ta	lg	69	f	0	1	0	0
38	cgi_030	urine	positive	ta	lg	69	f	0	1	1	1
38	cgi_029	tumor	positive	ta	lg	69	f	0	1	1	0
38	cgi_097	urine	negative	ta	lg	69	f	0	1	0	1
38	cgi_093	urine	negative	ta	lg	69	f	0	1	0	1
39	cgi_394	urine	negative			68	m	0	0	0	1
39	cgi_293	urine	negative	ta	lg	68	m	0	0	0	1
40	cgi_402	urine	negative			76	m	0	0	0	1
40	cgi_301	urine	negative			76	m	0	0	0	1
41	cgi_376	urine	positive	ta	lg	74	m	0	0	0	1
41	cgi_375	urine	positive	ta	lg	74	m	0	0	0	1
41	cgi_286	urine	positive	ta	lg	75	m	0	0	0	1
42	cgi_035	tumor	positive	t2	hg	75	f	1	1	1	0
42	cgi_033	urine	positive	t2	hg	75	f	1	1	1	1
42	cgi_034	urine	positive	t2	hg	75	f	1	1	0	1
43	cgi_037	tumor	positive	t1	hg	84	m	0	1	1	0
43	cgi_038	urine	positive	t1	hg	84	m	0	1	0	1
43	cgi_036	urine	positive	t1	hg	84	m	0	1	1	1
45	cgi_039	tumor	positive	ta	lg	69	f	0	1	1	0
45	cgi_040	urine	positive	ta	lg	69	f	0	1	1	1
45	cgi_096	urine	positive	pun-lmp	pun-lmp	69	f	0	1	0	0
46	cgi_043	urine	negative	t2	hg	90	m	1	1	0	0
46	cgi_041	tumor	positive	t2	hg	90	m	1	1	1	0
46	cgi_042	urine	positive	t2	hg	90	m	1	1	1	1
47	cgi_045	urine	positive	t1	hg	65	m	0	1	1	0
47	cgi_044	tumor	positive	t1	hg	65	m	0	1	1	0
47	cgi_046	urine	positive	t1	hg	65	m	0	1	0	1
48	cgi_217	urine	negative	t1	hg	80	f	0	0	0	1
48	cgi_215	urine	negative	t1	hg	81	f	0	0	0	1
48	cgi_209	urine	negative	t1	hg	80	f	0	0	0	1
48	cgi_208	urine	negative	t1	hg	80	f	0	0	0	1
48	cgi_206	urine	negative	t1	hg	80	f	0	0	0	1
49	cgi_304	urine	negative	ta	hg	80	m	0	0	0	1
49	cgi_323	urine	negative	ta	hg	80	m	0	0	0	1
50	cgi_326	urine	positive	ta	lg	86	m	0	0	0	1
51	cgi_327	urine	negative	t1	hg	59	m	0	0	0	1
51	cgi_262	urine	negative	t1	hg	58	m	0	0	0	1
52	cgi_265	urine	positive			67	m	0	0	0	1
52	cgi_334	urine	positive	ta	lg	67	m	0	0	0	1
53	cgi_346	urine	negative	t2	hg	71	m	0	0	0	1

53	cgi_294	urine	negative	t2	hg	71	m	0	0	0	1
54	cgi_384	urine	negative	t1	hg	69	m	0	0	0	1
54	cgi_271	urine	negative	t1	hg	69	m	0	0	0	1
56	cgi_386	urine	negative	t1	lg	49	m	0	0	0	1
56	cgi_285	urine	negative	t1	lg	49	m	0	0	0	1
56	cgi_284	urine	negative	t1	lg	49	m	0	0	0	1
57	cgi_202	urine	negative	ta	hg	85	f	0	0	0	1
57	cgi_214	urine	negative	ta	hg	86	f	0	0	0	1
58	cgi_256	urine	positive	ta	lg	88	m	0	0	0	1
58	cgi_246	urine	positive	t1	lg	86	m	0	0	0	1
59	cgi_175	urine	negative	ta	hg	87	m	0	0	0	1
59	cgi_173	urine	positive	ta	hg	87	m	0	0	0	1
60	cgi_230	urine	negative			54	f	0	0	0	1
60	cgi_224	urine	negative			54	f	0	0	0	1
61	cgi_154	urine	negative			76	m	0	0	0	1
61	cgi_171	urine	negative			76	m	0	0	0	1
62	cgi_047	tumor	positive	ta	lg	69	m	0	1	0	0
62	cgi_049	urine	negative	ta	lg	69	m	0	1	0	1
63	cgi_092	urine	negative	t2	hg	80	f	0	1	0	0
63	cgi_050	tumor	positive	t2	hg	80	f	0	1	0	0
64	cgi_051	tumor	positive	t1	hg	84	m	1	1	1	0
64	cgi_052	urine	positive	t1	hg	84	m	1	1	1	1
65	cgi_054	urine	positive	ta	hg	77	m	0	1	1	1
65	cgi_053	tumor	positive	ta	hg	77	m	0	1	1	0
66	cgi_095	urine	negative	t2	hg	78	m	0	1	0	0
66	cgi_055	tumor	positive	t2	hg	78	m	0	1	0	0
67	cgi_056	tumor	positive	ta	hg	63	m	0	1	1	0
67	cgi_058	urine	positive	ta	hg	63	m	0	1	1	1
68	cgi_059	urine	positive	ta	lg	72	m	0	1	1	1
68	cgi_060	tumor	positive	ta	lg	72	m	0	1	1	0
69	cgi_061	urine	positive	ta	lg	82	m	1	1	1	1
69	cgi_062	tumor	positive	ta	lg	82	m	1	1	1	0
70	cgi_064	tumor	positive	t1	hg	84	m	1	1	1	0
70	cgi_063	urine	positive	t1	hg	84	m	1	1	1	1
71	cgi_067	urine	negative	ta	lg	58	m	0	1	0	1
71	cgi_065	tumor	positive	ta	lg	58	m	0	1	0	0
71	cgi_066	urine	negative	Ta	lg	58	m	0	1	0	1
72	cgi_069	urine	positive	para-gangli-oma	para-gangli-oma	69	m	0	0	1	0
72	cgi_068	tumor	positive	para-gangli-oma	para-gangli-oma	69	m	0	0	1	0
73	cgi_205	urine	positive		hg	74	m	0	0	0	1
73	cgi_198	urine	positive	ta	hg	74	m	0	0	0	1
	cgi_099	urine	positive	t1	hg	57	m	0	0	0	1
	cgi_100	urine	positive	t1	hg		m	0	0	0	1
	cgi_101	urine	positive	t1	hg		m	0	0	0	1
	cgi_102	urine	positive	t1	hg		m	0	0	0	1

cgi_103	urine	positive	t1	hg		m	0	0	0	1
cgi_104	urine	positive	t4	hg		f	0	0	0	1
cgi_105	urine	negative				m	0	0	0	1
cgi_106	urine	positive	t2	hg		f	0	0	0	1
cgi_107	urine	positive	ta	hg		m	0	0	0	1
cgi_108	urine	positive	ta	hg		m	0	0	0	1
cgi_109	urine	positive	ta	lg	69	m	0	0	0	1
cgi_110	urine	negative			84	m	0	0	0	1
cgi_111	urine	positive	cis	hg	60	m	0	0	0	1
cgi_112	urine	positive	ta	hg		m	0	0	0	1
cgi_113	urine	positive	t1	hg		m	0	0	0	1
cgi_114	urine	positive	t2	hg		m	0	0	0	1
cgi_115	urine	positive	ta	lg		m	0	0	0	1
cgi_116	urine	negative	ta	lg		m	0	0	0	1
cgi_117	urine	positive	t1	hg		m	0	0	0	1
cgi_118	urine	positive	t4	hg		f	0	0	0	1
cgi_119	urine	positive	t2	hg		f	0	0	0	1
cgi_120	urine	positive	t2	hg		m	0	0	0	1
cgi_121	urine	positive		hg		m	0	0	0	1
cgi_124	urine	positive	ta	lg	67	m	0	0	0	1
cgi_125	urine	negative			81	m	0	0	0	1
cgi_126	urine	positive	t2	hg	75	m	0	0	0	1
cgi_129	urine	negative	ta,cis	hg	66	m	0	0	0	1
cgi_135	urine	negative	ta	lg	72	m	0	0	0	1
cgi_137	urine	positive	ta	lg	56	f	0	0	0	1
cgi_138	urine	negative	t1	lg	69	m	0	0	0	1
cgi_139	urine	negative	t1	hg	89	m	0	0	0	1
cgi_140	urine	negative	ta	lg	78	m	0	0	0	1
cgi_142	urine	positive			72	m	0	0	0	1
cgi_144	urine	negative	ta	lg	59	m	0	0	0	1
cgi_145	urine	negative	ta	lg	70	m	0	0	0	1
cgi_147	urine	negative	ta	lg	84	f	0	0	0	1
cgi_148	urine	negative	ta	lg	67	f	0	0	0	1
cgi_149	urine	negative			20	f	0	0	0	1
cgi_150	urine	negative			72	m	0	0	0	1
cgi_151	urine	negative			32	m	0	0	0	1
cgi_152	urine	negative	ta	lg	72	m	0	0	0	1
cgi_153	urine	negative			62	f	0	0	0	1
cgi_155	urine	negative			87	m	0	0	0	1
cgi_156	urine	negative	ta	lg	71	m	0	0	0	1
cgi_157	urine	negative			64	m	0	0	0	1
cgi_159	urine	negative			89	m	0	0	0	1
cgi_160	urine	negative	t1	lg	72	m	0	0	0	1
cgi_162	urine	negative			85	f	0	0	0	1
cgi_163	urine	negative			90	m	0	0	0	1
cgi_164	urine	negative			84	f	0	0	0	1
cgi_165	urine	negative			57	m	0	0	0	1
cgi_168	urine	negative			77	f	0	0	0	1
cgi_169	urine	negative			81	f	0	0	0	1

cgi_170	urine	negative	t3	hg	77	m	0	0	0	1
cgi_174	urine	negative		hg	75	f	0	0	0	1
cgi_176	urine	negative	ta	lg	74	f	0	0	0	1
cgi_177	urine	negative	ta	lg	89	m	0	0	0	1
cgi_178	urine	negative			92	f	0	0	0	1
cgi_179	urine	negative	ta	lg	78	f	0	0	0	1
cgi_180	urine	negative	t1	lg	76	m	0	0	0	1
cgi_181	urine	negative	ta	hg	81	m	0	0	0	1
cgi_182	urine	positive	ta	lg	72	m	0	0	0	1
cgi_184	urine	positive	ta	lg	71	m	0	0	0	1
cgi_186	urine	positive	t4	hg		m	0	0	0	1
cgi_187	urine	negative			34	m	0	0	0	1
cgi_188	urine	negative			22	f	0	0	0	1
cgi_189	urine	negative			64	m	0	0	0	1
cgi_190	urine	negative			55	m	0	0	0	1
cgi_191	urine	negative			83	m	0	0	0	1
cgi_192	urine	positive	ta	lg	68	m	0	0	0	1
cgi_193	urine	negative			67	m	0	0	0	1
cgi_196	urine	negative			82	m	0	0	0	1
cgi_197	urine	negative			69	f	0	0	0	1
cgi_199	urine	negative	ta	lg	77	f	0	0	0	1
cgi_200	urine	negative			68	m	0	0	0	1
cgi_201	urine	negative			81	f	0	0	0	1
cgi_203	urine	negative			83	f	0	0	0	1
cgi_204	urine	negative			86	m	0	0	0	1
cgi_211	urine	negative	ta	lg	73	f	0	0	0	1
cgi_219	urine	negative			80	f	0	0	0	1
cgi_221	urine	negative			62	m	0	0	0	1
cgi_222	urine	negative			73	m	0	0	0	1
cgi_223	urine	negative			60	m	0	0	0	1
cgi_225	urine	negative			80	m	0	0	0	1
cgi_226	urine	negative			64	m	0	0	0	1
cgi_227	urine	negative			56	m	0	0	0	1
cgi_228	urine	negative			56	m	0	0	0	1
cgi_229	urine	positive	ta	hg	92	f	0	0	0	1
cgi_231	urine	negative			39	f	0	0	0	1
cgi_232	urine	negative			71	m	0	0	0	1
cgi_233	urine	negative			28	f	0	0	0	1
cgi_237	urine	positive	t3	hg	66	m	0	0	0	1
cgi_238	urine	positive		hg	94	m	0	0	0	1
cgi_239	urine	negative			73	m	0	0	0	1
cgi_240	urine	negative			55	f	0	0	0	1
cgi_241	urine	positive	t2	hg	67	m	0	0	0	1
cgi_242	urine	negative				unknown	0	0	0	1
cgi_243	urine	positive	ta	lg	84	m	0	0	0	1
cgi_244	urine	negative			65	f	0	0	0	1
cgi_257	urine	positive	ta	hg	84	m	0	0	0	1

cgi_258	urine	positive	ta,cis	hg	60	m	0	0	0	1
cgi_259	urine	negative			66	f	0	0	0	1
cgi_260	urine	negative			35	m	0	0	0	1
cgi_261	urine	negative			78	m	0	0	0	1
cgi_263	urine	negative			88	f	0	0	0	1
cgi_267	urine	negative	ta	lg	76	m	0	0	0	1
cgi_268	urine	negative	ta,cis	hg	72	m	0	0	0	1
cgi_272	urine	negative	ta	lg	57	m	0	0	0	1
cgi_273	urine	negative			71	f	0	0	0	1
cgi_274	urine	negative			45	f	0	0	0	1
cgi_276	urine	negative	ta	hg	54	m	0	0	0	1
cgi_278	urine	negative			80	f	0	0	0	1
cgi_279	urine	negative	ta	lg	59	m	0	0	0	1
cgi_280	urine	negative			44	f	0	0	0	1
cgi_281	urine	negative	ta	lg	71	m	0	0	0	1
cgi_282	urine	negative	t1	hg			0	0	0	1
cgi_283	urine	negative			65	m	0	0	0	1
cgi_287	urine	negative			44	f	0	0	0	1
cgi_288	urine	negative	ta	lg	66	m	0	0	0	1
cgi_289	urine	negative			80	f	0	0	0	1
cgi_291	urine	negative	ta	lg	68	m	0	0	0	1
cgi_292	urine	negative	t1	hg	85	m	0	0	0	1
cgi_295	urine	negative	ta	lg	79	m	0	0	0	1
cgi_296	urine	negative	ta	lg	73	m	0	0	0	1
cgi_297	urine	negative	t1	hg	80	m	0	0	0	1
cgi_298	urine	negative			74	m	0	0	0	1
cgi_299	urine	negative	ta	lg	65	m	0	0	0	1
cgi_300	urine	negative			64	f	0	0	0	1
cgi_303	urine	positive	ta	lg	79	f	0	0	0	1
cgi_305	urine	negative			81	f	0	0	0	1
cgi_306	urine	negative			51	f	0	0	0	1
cgi_307	urine	negative			68	f	0	0	0	1
cgi_308	urine	negative	ta	lg	66	m	0	0	0	1
cgi_309	urine	positive	ta	lg	50	m	0	0	0	1
cgi_310	urine	negative			84	m	0	0	0	1
cgi_311	urine	negative			48	m	0	0	0	1
cgi_312	urine	negative	ta	hg	64	m	0	0	0	1
cgi_313	urine	negative	t1	hg	80	m	0	0	0	1
cgi_314	urine	positive			86	m	0	0	0	1
cgi_315	urine	negative			18	f	0	0	0	1
cgi_316	urine	negative			46	m	0	0	0	1
cgi_317	urine	negative	ta	lg	49	f	0	0	0	1
cgi_318	urine	negative			47	f	0	0	0	1
cgi_319	urine	positive	cis	hg	83	m	0	0	0	1
cgi_320	urine	negative			70	m	0	0	0	1
cgi_321	urine	negative			39	m	0	0	0	1
cgi_324	urine	negative	t1	hg	80	m	0	0	0	1
cgi_325	urine	negative			62	m	0	0	0	1
cgi_328	urine	negative			70	m	0	0	0	1

cgi_329	urine	positive	ta	lg	84	f	0	0	0	1
cgi_330	urine	negative			82	f	0	0	0	1
cgi_331	urine	negative			41	m	0	0	0	1
cgi_333	urine	negative	ta	lg	80	f	0	0	0	1
cgi_335	urine	negative			60	f	0	0	0	1
cgi_336	urine	negative			59	m	0	0	0	1
cgi_340	urine	negative			72	f	0	0	0	1
cgi_341	urine	negative	ta	lg	80	m	0	0	0	1
cgi_342	urine	negative			61	m	0	0	0	1
cgi_343	urine	negative	ta	lg	79	f	0	0	0	1
cgi_344	urine	positive	ta	lg	78	m	0	0	0	1
cgi_345	urine	negative			77	m	0	0	0	1
cgi_347	urine	positive	t2	hg	80	f	0	0	0	1
cgi_348	urine	negative			43	f	0	0	0	1
cgi_349	urine	negative			39	m	0	0	0	1
cgi_351	urine	negative			69	m	0	0	0	1
cgi_352	urine	negative			38	f	0	0	0	1
cgi_353	urine	negative			70	m	0	0	0	1
cgi_354	urine	negative	t1	hg	79	m	0	0	0	1
cgi_355	urine	negative	ta	hg	65	m	0	0	0	1
cgi_356	urine	negative			26	f	0	0	0	1
cgi_357	urine	negative	t1	hg	77	m	0	0	0	1
cgi_358	urine	negative			72	f	0	0	0	1
cgi_359	urine	negative			45	m	0	0	0	1
cgi_360	urine	negative			86	m	0	0	0	1
cgi_361	urine	negative			21	f	0	0	0	1
cgi_362	urine	negative			26	m	0	0	0	1
cgi_363	urine	negative			51	f	0	0	0	1
cgi_365	urine	negative			80	f	0	0	0	1
cgi_366	urine	positive	ta	lg	75	m	0	0	0	1
cgi_367	urine	negative	ta	hg	67	m	0	0	0	1
cgi_368	urine	negative			41	m	0	0	0	1
cgi_369	urine	negative			73	m	0	0	0	1
cgi_370	urine	negative	ta	hg	76	m	0	0	0	1
cgi_371	urine	negative			47	m	0	0	0	1
cgi_372	urine	negative			28	m	0	0	0	1
cgi_373	urine	negative			38	f	0	0	0	1
cgi_374	urine	negative			41	m	0	0	0	1
cgi_377	urine	negative			61	m	0	0	0	1
cgi_378	urine	negative			85	f	0	0	0	1
cgi_379	urine	negative	ta	lg	65	m	0	0	0	1
cgi_380	urine	positive	t1	hg	84	m	0	0	0	1
cgi_382	urine	positive	ta	lg	77	f	0	0	0	1
cgi_383	urine	negative	ta	lg	82	m	0	0	0	1
cgi_387	urine	negative	t1	hg	78	m	0	0	0	1
cgi_388	urine	negative	ta	lg	56	m	0	0	0	1
cgi_390	urine	negative	ta	lg	49	m	0	0	0	1
cgi_392	urine	negative	t1	hg	78	m	0	0	0	1
cgi_395	urine	negative	ta	lg	84	f	0	0	0	1

cgi_398	urine	negative	ta	lg	69	m	0	0	0	1
cgi_399	urine	positive	ta	lg	76	m	0	0	0	1
cgi_400	urine	negative			71	f	0	0	0	1
cgi_401	urine	negative	t1	hg	75	f	0	0	0	1
cgi_403	urine	negative	t1	hg	66	m	0	0	0	1
cgi_407	urine	negative	ta	lg	73	m	0	0	0	1
cgi_408	urine	negative	ta	hg	75	m	0	0	0	1
cgi_409	urine	negative			72	m	0	0	0	1
cgi_410	urine	negative	ta	lg	71	m	0	0	0	1
cgi_411	urine	negative	t1	hg	63	m	0	0	0	1
cgi_412	urine	negative				f	0	0	0	1
cgi_413	urine	negative				f	0	0	0	1
cgi_414	urine	negative				m	0	0	0	1
cgi_415	urine	negative				f	0	0	0	1
cgi_416	urine	negative				f	0	0	0	1
cgi_417	urine	negative				f	0	0	0	1
cgi_418	urine	negative				f	0	0	0	1
cgi_419	urine	negative				m	0	0	0	1
cgi_420	urine	negative				m	0	0	0	1
cgi_421	urine	negative				m	0	0	0	1
cgi_422	urine	negative			71	m	0	0	0	1
cgi_423	urine	negative			76	m	0	0	0	1
cgi_424	urine	negative			64	f	0	0	0	1
cgi_425	urine	negative			49	f	0	0	0	1
cgi_426	urine	negative			78	m	0	0	0	1
cgi_427	urine	negative			40	f	0	0	0	1
cgi_428	urine	negative			36	m	0	0	0	1
cgi_429	urine	negative			80	f	0	0	0	1
cgi_430	urine	negative			90	m	0	0	0	1
cgi_431	urine	negative			30	f	0	0	0	1
cgi_432	urine	negative			28	m	0	0	0	1
cgi_433	urine	negative			37	m	0	0	0	1
cgi_434	urine	negative			56	f	0	0	0	1
cgi_435	urine	negative			62	f	0	0	0	1
cgi_436	urine	negative			60	m	0	0	0	1
cgi_437	urine	negative			35	m	0	0	0	1
cgi_439	urine	negative			59	f	0	0	0	1
cgi_440	urine	positive	t2	hg		m	0	0	0	1
cgi_441	urine	negative			74	f	0	0	0	1
cgi_442	urine	negative			73	f	0	0	0	1
cgi_443	urine	negative			29	f	0	0	0	1
cgi_444	urine	negative			45	m	0	0	0	1
cgi_445	urine	positive	ta	lg	88	m	0	0	0	1
cgi_446	urine	negative			68	f	0	0	0	1
cgi_447	urine	negative			65	m	0	0	0	1
cgi_448	urine	negative			56	m	0	0	0	1
cgi_449	urine	negative			57	f	0	0	0	1
cgi_450	urine	negative			57	m	0	0	0	1
cgi_451	urine	negative			35	f	0	0	0	1

cgi_452	urine	negative			58	m	0	0	0	1
cgi_453	urine	negative			38	f	0	0	0	1
cgi_454	urine	negative			72	m	0	0	0	1
cgi_455	urine	negative			59	f	0	0	0	1
cgi_456	urine	negative			58	m	0	0	0	1
cgi_457	urine	negative			75	m	0	0	0	1
cgi_458	urine	negative			72	m	0	0	0	1
cgi_459	urine	negative			77	m	0	0	0	1
cgi_460	urine	negative			59	f	0	0	0	1
cgi_461	urine	negative			18	f	0	0	0	1
cgi_462	urine	negative			62	m	0	0	0	1
cgi_463	urine	negative			60	m	0	0	0	1
cgi_464	urine	negative			69	m	0	0	0	1
cgi_465	urine	positive	t2	hg		m	0	0	0	1
cgi_466	urine	positive	ta	hg		m	0	0	0	1
cgi_467	urine	positive	ta	lg		f	0	0	0	1
cgi_468	urine	positive	cis	hg		m	0	0	0	1
cgi_469	urine	positive	t2	hg		m	0	0	0	1
cgi_470	urine	positive	t1	hg		m	0	0	0	1
cgi_471	urine	positive	t2	hg		f	0	0	0	1
cgi_472	urine	positive	ta	lg		f	0	0	0	1
cgi_473	urine	positive	ta	hg		m	0	0	0	1
cgi_474	urine	positive	t2	hg		m	0	0	0	1
cgi_475	urine	positive	t4	hg	84	f	0	0	0	1
cgi_476	urine	positive	ta	lg	81	m	0	0	0	1
cgi_477	urine	positive	t3	hg	73	m	0	0	0	1
cgi_478	urine	positive	t1	hg	77	f	0	0	0	1
cgi_479	urine	positive	t1	hg	77	f	0	0	0	1
cgi_480	urine	positive	t1	hg	90	f	0	0	0	1
